# Graves‘ disease following vaccination against SARS-CoV-2: A systematic review of the reported cases

**DOI:** 10.3389/fendo.2022.938001

**Published:** 2022-09-27

**Authors:** Konstantinos Katsikas Triantafyllidis, Panagiotis Giannos, Dimitra Stathi, Konstantinos S. Kechagias

**Affiliations:** ^1^ Society of Meta-research and Biomedical Innovation, London, United Kingdom; ^2^ Department of Nutrition and Dietetics, Homerton University Hospital Foundation Trust, London, United Kingdom; ^3^ Department of Life Sciences, Faculty of Natural Sciences, Imperial College London, London, United Kingdom; ^4^ Department of Endocrinology and Diabetes, Guy’s and St Thomas’ National Health Service (NHS) Foundation Trust, London, United Kingdom; ^5^ Department of Metabolism, Digestion and Reproduction, Faculty of Medicine, Imperial College London, London, United Kingdom

**Keywords:** Graves’ disease, thyroiditis, COVID-19, SARS–CoV–2, vaccines

## Abstract

The newly developed COVID-19 vaccines have established a safe profile, yet some individuals experience a wide range of adverse events. Recently, thyroid dysfunction, including Graves’ disease, has been observed after administration of different COVID-19 vaccines, although causality remains a matter of debate. The aim of this systematic review was to examine the available literature and provide an overview of reported cases of Graves’ disease following COVID-19 vaccination. We identified 21 eligible articles which included 57 patients with Graves’ disease following COVID-19 vaccination. Fourteen participants were males (25%, 14/57) and 43 (75%, 44/57) were females with a mean age of 44.3 years. The most common presenting symptom was palpitations (63%, 27/43) followed by weight loss (35%, 15/43). The majority of patients received thionamides (47%, 25/53). The clinical status after treatment was provided for 37 patients and it was improved in the majority of them (84%, 31/37). Graves’ disease is possibly a condition clinicians may expect to encounter in patients receiving COVID-19 vaccines. While the above adverse event is rare, considering the scarcity of available data in scientific literature, and causality is not yet confirmed, the increased awareness of clinicians and the early recognition of the disorder are important for the optimal management of these patients.

## Introduction

An outbreak of an atypical viral pneumonia initially reported at the end of 2019, was later declared a public health emergency of international concern in March 2020 (1, 2). The aetiology was a novel coronavirus strain called Severe Acute Respiratory Syndrome Coronavirus 2 (SARS-CoV-2), the cause of coronavirus disease 2019 (COVID-19), which has now disseminated across the globe with hundreds of millions affected (3, 4).

Different vaccines have been used widely against COVID-19 including: COMIRNATY (the COVID-19 mRNA vaccine BNT162b2 by BioNTech–Pfizer); COVID-19 Vaccine Moderna (mRNA-1273 by Moderna); VAXZEVRIA (ChAdOx1-nCoV19 by AstraZeneca-Oxford University); COVID-19 Vaccine Janssen (Ad26.COV2.S by Janssen); and CoronaVac COVID19 vaccine (Vero cell by Sinovac Biotech) (5, 6). Almost two thirds of the world population has now received at least one dose of a COVID-19 vaccine with 12 billion doses already administered worldwide (7).

Time has proven the aforementioned vaccines both safe and effective, with serious adverse events being rare, while providing 70-95% protection against severe disease (8–11). However, adverse reactions following vaccination remain inevitable, considering the extent and scale required to control seasonal outbreaks of COVID-19 infection (12–14). At present, patients experience numerous commonly reported adverse symptoms following COVID-19 vaccination, including muscle pain, fever, headache, nausea and vomiting. Beyond the most commonly presenting adverse effects post-COVID-19 vaccination, a diverse range of complaints and symptoms have been reported by patients, including also cases of immune-mediated adverse events (12–17). More recently though, there is an increasing number of reports pertained to thyroid disorders described in patients after the first or second doses of COVID-19 vaccination; however, they are not yet fully clarified.

Recent evidence suggests that viral effects of COVID-19 infection might be associated with thyroid function, possibly by contributing to the onset of thyroid disease or to the exacerbation of a pre-existing one (18–20). To date, COVID-19 vaccine administration has not been considered as a precipitating factor of thyroid dysfunction. In this study, we comprehensively examined the currently available literature to provide an overview of the reported cases of Graves’ disease following vaccination against SARS-CoV-2.

## Methods

This review was reported based on the “Preferred Reporting Items for Systematic Reviews and Meta-Analyses” (PRISMA) guidelines.

### Literature search

Two reviewers (KKT, PG) searched PubMed and Scopus library databases from inception until May 2022 independently. The search included the following terms: “(COVID 19 vaccin* OR SARS-COV2 vaccin*) AND (Graves’ disease OR Basedow Disease OR Exophthalmic Goiter OR Thyroiditis)”. No restrictions regarding study design, geographic region or language were applied. A manual search of references cited in the selected articles and published reviews were also ensued for undetected studies. Discrepancies in the literature search process were resolved by a third investigator (KSK).

### Eligibility criteria

We included studies that provided data for new onset or exacerbation of Graves’ disease following COVID-19 vaccination with at least one dose. All study designs were considered eligible for inclusion. Review articles, abstracts submitted in conferences and non-peer reviewed sources were not eligible for inclusion. Studies on *in vitro* and animal models were excluded.

### Data extraction and handling

In all studies, patient data was retrieved and handled by two authors (KKT, PG) who conducted the data extraction independently. We collected the following information: sex, age, comorbidities, type of vaccine, number of doses received, presenting symptoms after vaccination, history of COVID-19 infection, laboratory measurements, primary diagnosis, imaging findings, treatment, clinical outcome. Any disagreements were discussed and resolved by a third author (KSK).

### Quality assessment

The studies were evaluated using the criteria established by the Task Force for Reporting Adverse Events of the International Society for Pharmacoepidemiology (ISPE) and the International Society of Pharmacovigilance (ISoP) (21). The assessment was based on the adequate reporting of 12 different elements namely: title, patient demographics, current health status, medical history, physical examination, patient disposition, drug identification, dosage, administration/drug reaction interface, concomitant therapies, adverse events, and discussion. The studies scored either 0 (absence of information) or 1 (containing the information) for every element.

## Results

### Study characteristics

**Figure 1:**
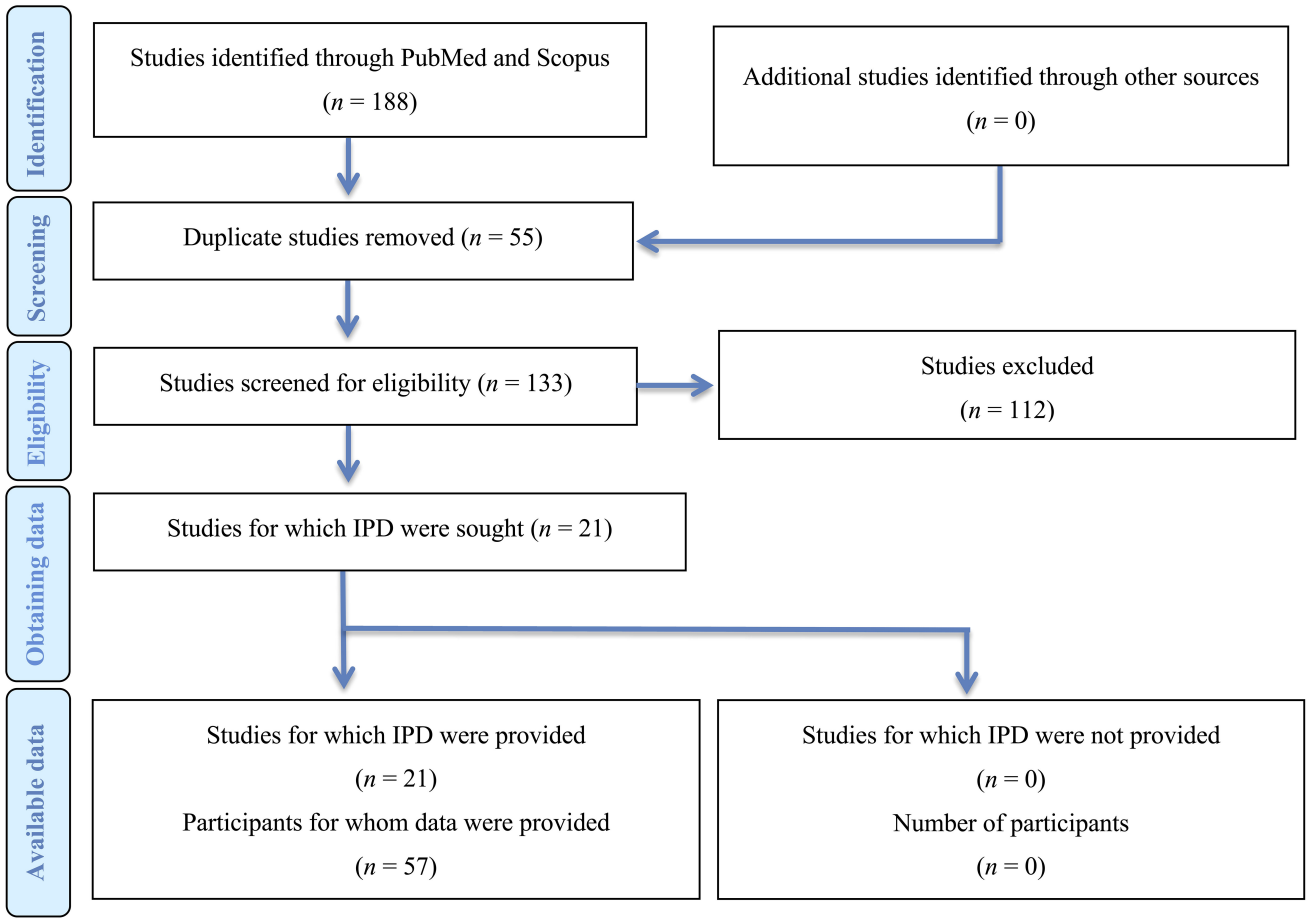
Prisma flowchart.

The initial literature search yielded 188 publications. In the first screening 165 studies were excluded as irrelevant. After the exclusion phase, 21 studies (22–42) were eligible for the systematic review (**Figure 1**). Ten of the studies were conducted in Asia, 6 in Europe, 4 in Americas, and 1 in Australia. In terms of design, 12 studies were case series and 9 were case reports (**Table 1**).

**Table 1:** Characteristics of the included studies.

Author,Year,Country	Case number	Age and Gender	Comorbidities	Previousthyroid disease(medications)	PreviousCOVID-19 infection	COVID-19 vaccine type and dose	New onset/relapse of Graves’ disease post vaccination	Main presenting symptoms	Days for the onset of symptoms	Treatment	Outcome
Bostan, 2022 (38), Turkey	Case 1	44 F	No	Graves’ Disease	No	CoronaVac 1^st^ dose	Relapse	• Sweating • Palpitation • Fatigue	7	Methimazole, Propranolol	NA
	Case 2	49 M	No	Graves’ Disease	No	COMIRNATY 2^nd^ dose	Relapse	• Sweating • Palpitations • Tremor	30	Methimazole, Propranolol	Improvement after 4 weeks
	Case 3	31 F	Breast cancer	Graves’ Disease	No	COMIRNATY 1^st^ dose	Relapse	• Sweating • Hot flushes • Weakness	21	Methimazole, Propranolol	Improvement after 5 weeks
	Case 4	53 F	No	Hashimoto’s thyroiditis (On levothyroxine)	Yes	COMIRNATY 1^st^ dose	New onset	• Sweating • Palpitations • Weight loss	7	Propranolol	Improvement after 8 weeks
	Case 5	51 F	Diabetes, Hypertension	No	NA	COMIRNATY 2^nd^ dose	New onset	• Right eye proptosis • Irritation • Dryness	4	Methimazole, Propranolol	Thyroidectomy after 4 months
	Case 6	47 F	Obesity	No	No	COMIRNATY 1^st^ dose	New onset	• Sweating • Palpitations	5	Methimazole, Propranolol	Improvement after 4 weeks
	Case 7	46 M	No	No	No	COMIRNATY 2^nd^ dose	New onset	• Sweating • Emotional liability • Palpitations • Weight loss	21	Methimazole, Propranolol	Improvement after 4 weeks
Chee, 2022 (39), Singapore	Case 1	33 F	NA	No	No	mRNA vaccine* 1st dose	New onset	NA	7	Carbimazole, Propranolol	Improvement after 4 weeks
	Case 2	37 F	NA	No	No	mRNA vaccine* 1st dose	New onset	NA	7	Carbimazole, Propranolol	Improvement after 32 days
	Case 3	37 F	NA	No	No	mRNA vaccine* 2nd dose	New onset	NA	21	Carbimazole, Propranolol	Improvement after 53 days
	Case 4	34 F	NA	No	No	mRNA vaccine* 1st dose	New onset	NA	26	Carbimazole, Propranolol	Improvement after 58 days
	Case 5	33 F	NA	No	No	mRNA vaccine* 2nd dose	New onset	NA	9	Carbimazole, Propranolol	Improvement after 64 days
	Case 6	43 F	NA	No	No	mRNA vaccine* 2nd dose	New onset	NA	13	Carbimazole	Improvement after 29 days
	Case 7	59 M	NA	Graves’ Disease	No	mRNA vaccine* 1st dose	Relapse	NA	21	Carbimazole	Still not in remission
	Case 8	74 F	NA	Graves’ Disease	No	mRNA vaccine* 2nd dose	Relapse	•Asymptomatic	11	Carbimazole	NA
	Case 9	25 F	NA	Graves’ Disease	No	mRNA vaccine* 2nd dose	Relapse	•Asymptomatic	31	Carbimazole	Improvement after 123 days
	Case 10	41 F	NA	Graves’ Disease	No	mRNA vaccine* 2nd dose	Relapse	NA	28	Carbimazole	Improvement after 31 days
	Case 11	24 F	NA	Graves’ Disease	No	mRNA vaccine* 2nd dose	Relapse	•Asymptomatic	63	Carbimazole	Improvement after 42 days
	Case 12	22 F	NA	Graves’ Disease	No	mRNA vaccine* 1st dose	Relapse	NA	5	Carbimazole, Propranolol	Improvement after 178 days
Chua, 2022 (37), Singapore	Case 1	41 M	NA	Graves’ Disease (On carbimazole)	NA	COVID-19 Vaccine Moderna 1^st^ dose	Relapse	•Tremor •Palpitations	5	Carbimazole	NA
	Case 2	45 F	NA	No	NA	COMIRNATY 1^st^ dose	New onset	•Chest tightness •Palpitations	4	Carbimazole	NA
Di Fillipo, 2021 (35), Italy	Case 1	32 M	No	No	NA	VAXZEVRIA 2^nd^ dose	New onset	•Anxiety •Tachycardia •Palpitations	10	Propranolol, Thiamazole, Propylthiouracil (switched from thiamazole)	Improvement after 3 months
	Case 2	35 M	No	No	NA	VAXZEVRIA 1^st^ dose	New onset	•Headache •Nausea •Asthenia •Palpitations •Tachycardia •Opthalmopathy	5	Thiamazole, Propranolol	Improvement after 3 months
Goblirsch, 2021, (23) USA	Case 1	71 F	Breast cancer, Struma ovarii	Multinodular goitre	No	COMIRNATY 2^nd^ dose	New onset	•Palpitations •Fever •Sweating •Dyspnoea •Leg swelling •Dizziness •Nausea •Diarrhoea •Abdominal pain •Tremor	14	Methimazole, Atenolol	Improvement of symptoms but moderate to severe Graves opthalmopathy
Hamouche, 2021, (25)	Case 1	32 M	No	No	Yes	COMIRNATY 1^st^ dose	New onset	•Dry cough •Low-grade fever •Fatigue •Palpitations •Insomnia •Tremor •Irritability •Diaphoresis •Dyspnoea	10	Methimazole, Propranolol, Prednisone	Improvement after 6 weeks
Lee, 2021, (41) South Korea	Case 1	46 F	NA	NA	NA	VAXZEVRIA 1st dose	New onset	•Chest pain •Dyspnoea	1	NA	NA
	Case 2	73 F	NA	NA	NA	VAXZEVRIA 2^nd^ dose	New onset	•Weight loss •Dyspnoea	14	NA	NA
	Case 3	39 M	NA	Graves’ Disease	NA	COVID-19 Vaccine Janssen 1^st^ dose	New onset	•Fever •Neck pain	14	NA	NA
	Case 4	34 M	NA	NA	NA	COVID-19 Vaccine Janssen 1^st^ dose	NA	•Weight loss •Palpitations	14	NA	NA
Lui, 2021, (26) China	Case 1	32 F	No	Hypothyroidism (On thyroxine)	No	COMIRNATY 2^nd^ dose	New onset	•Palpitations	38	Carbimazole, Propranolol	Improvement
Oguz, 2021, (36) Turkey	Case 1	40 F	No	No	NA	COMIRNATY 3^rd^ dose	New onset	NA	2	Methimazole	Not in remission yet
	Case 2	29 M	No	No	NA	COMIRNATY 1^st^ dose	New onset	NA	15	Nil	Improvement after 10 weeks
	Case 3	43 F	Ankylosing spondilitis	Multinodular goiter	NA	COMIRNATY 3^rd^ dose	New onset	NA	9	Methimazole	Not in remission yet
	Case 4	43 F	Diabetes insipidus	Autoimmune thyroiditis	NA	COMIRNATY 1^st^ dose	New onset	NA	14	Discontinue Levothyroxine	Hypothyroidism
	Case 5	34 F	No	No	NA	CoronaVac 1^st^ dose	New onset	NA	150	Methimazole, Prednisolone	Not in remission
Patrizio, 2021, (30) Italy	Case 1	52 M	Diabetes mellitus, Vitiligo vulgaris	No	No	COMIRNATY 2^nd^ dose	New onset	•Weight loss •Fatigue	28	Methimazole, Atenolol, Insulin analogues	Improvement
Pierman, 2021, (29) Belgium	Case 1	34 F	NA	Graves’ disease (On thiamazole)	NA	COMIRNATY 1^st^ dose	Relapse	•Ophthalmopathy •Tremor •Sweating •Thermophobia •Dyspnoea •Weight loss	10	Thiamazole	NA
Pla Peris, 2022, (22) Spain	Case 1	71 F	NA	NA	NA	COMIRNATY	NA	•Weight loss •Fatigue •Atrial fibrillation	60	Methimazole	NA
	Case 2	42 F	NA	NA	NA	COMIRNATY	NA	•Weight loss •Fatigue •Palpitations	10	Methimazole	NA
	Case 3	54 F	NA	NA	NA	COVID-19 Vaccine Moderna	NA	•Weight loss •Fatigue •Palpitations	10	Methimazole	NA
	Case 4	46 F	NA	NA	NA	COMIRNATY	NA	•Weight loss •Fatigue •Palpitations •Irritability	50	Methimazole	NA
	Case 5	69 F	NA	NA	NA	COMIRNATY	NA	•Neck pain •Fever •Weight loss •Palpitations •Tremor	10	Methimazole, NSAID	NA
Pujol, 2021, (27) Spain	Case 1	38 F	Mental retardation, Schizophrenia	No	NA	COMIRNATY 1^st^ dose	New onset	•Irritation •Insomnia •Sweating	12	Methimazole	NA
Raven, 2021, (40) Australia	Case 1	35 F	NA	No	NA	VAXZEVRIA 1^st^ dose	New onset	•Tremor •Palpitations •Hyperphagia •Thermophobia	5	Carbimazole	NA
Shih, 2022, (42) Taiwan	Case 1	39 F	NA	No	NA	COVID-19 Vaccine Moderna	New onset	•Tremor •Palpitations	14	Carbimazole	NA
	Case 2	59 F	NA	No	NA	VAXZEVRIA	New onset	•Dizziness •Palpitations	14	Carbimazole	NA
	Case 3	44 F	NA	No	NA	VAXZEVRIA	New onset	•Tremor •Thermophobia •Weight loss	4	Carbimazole	NA
Sriphrapradang, 2021 (I), (31) Thailand	Case 1	70 M	NA	NA	No	VAXZEVRIA 2^nd^ dose	NA	•Dyspnoea •Myalgia •Palpitations •Poor appetite •Weight loss	2	Methimazole	NA
Sriphrapradang, 2021 (II), (32) Thailand	Case 1	30 F	NA	Graves’ Disease (On methimazole)	NA	VAXZEVRIA 3^rd^ dose	Exacerbation	•Palpitations •Weight loss	4	Methimazole	Improvement after 30 days
Vera- Lastra, 2021, (34) Mexico	Case 1	40 F	Hypertension	No	NA	COMIRNATY	New onset	•Nausea •Vomiting •Fatigue •Insomnia •Palpitations	2	Thiamazole, Diltiazem, Ivabradine	Improvement
	Case 2	28 F	No	No	NA	COMIRNATY	New onset	•Anxiety •Insomnia •Palpitations •Tremor	3	Thiamazole, Propranolol	Improvement
Weintraub, 2021, (24) USA	Case 1	38 F	NA	NA	NA	COMIRNATY 1^st^ dose	New onset	•Tachycardia •Fever •Abdominal pain	5	Methimazole, Propranolol	Improvement after 3 months
	Case 2	63 F	NA	NA	NA	COVID-19 Vaccine Moderna 1^st^ dose	New onset	•Pruritic rash	7	Nil	Improvement
	Case 3	30 M	NA	NA	NA	COMIRNATY 2^nd^ dose	New onset	•Weight loss •Palpitations •Tremor •Irritability	28	Methimazole, Atenolol	Improvement after 6 weeks
Yamamoto, 2021, (28), Japan	Case 1	64 F	Colorectal cancer, Diabetes mellitus, Obesity	NA	No	COMIRNATY 1^st^ dose	New onset	•Fever •Fatigue •Dyspnoe •Decreased urine output •Leg swelling •Palpitations	6	Thiamazole, Potassium iodine, Corticosteroids, Furosemide, Carvedilol	Improvement after 11 days
Zettinig, 2021, (33) Austria	Case 1	71 F	Hemithyroidectomy	Grave’s disease	NA	COMIRNATY 2^nd^ dose	Relapse	•Palpitations	NA	Thyreostatic treatment	Improvement
	Case 2	46 M	NA	No	NA	COMIRNATY 1^st^ dose	New onset	•Asymptomatic	35	Thyreostatic treatment	Improvement

F, Female; M, Male; NA, Not available; NSAID, Non-steroidal anti-inflammatory drugs.

*Brand not specified.

We identified a total of 57 cases of Graves’ disease following COVID-19 vaccination. Fourteen participants were males (25%, 14/57) and 43 (75%, 43/57) were females with a mean age of 44.3 years (median: 41.5, interquartile range: 34-51.5). Data regarding medical history was provided for 30 cases and half of them had no past medical history (50%, 15/30) with two patients having hypothyroidism before vaccination (66%, 2/30). From the included patients 37 (74%, 37/50) were characterised as new-onset, 12 (26%, 12/50) as relapse and one (2%, 2/50) as exacerbation. The mean age of individuals with Graves’ disease relapse was 42.9 (median: 41, interquartile range: 28-59) with the majority of them receiving mRNA vaccines (92%, 11/12).

For most of the patients (58%, 33/57) data regarding COVID-19 infection before or at the time of Graves’ diagnosis was not provided. Among the remaining patients only 2 were previously infected with SARS-CoV-2. In 12 patients, vaccine brand was not mentioned (21%, 12/57). The majority of the patients received COMIRNATY (64%, 29/45), followed by VAXZEVRIA (18%, 8/45), while a fraction of participants received COVID-19 Vaccine Moderna (9%, 4/45), COVID-19 Vaccine Janssen (4%, 2/45) and CoronaVac (4%, 2/45).

Data regarding the day of the onset of symptoms was provided for 56 cases. On average, the symptoms developed 14.8 days (median: 10, interquartile range: 5-21) after the administration of the vaccine irrespective of the dose. A significant proportion of patients developed symptoms after the 1^st^ dose (55%, 26/47), followed by the 2^nd^ dose (38%, 18/47). Only 3 cases (6%, 3/47) developed symptoms after the 3^rd^ dose.

Data regarding symptomatology was provided for 43 cases. The most common symptom was palpitations (63%, 27/43) followed by weight loss (35%, 15/43). Other common symptoms included tremor (25%, 11/43) and fatigue/weakness (23%, 10/43). Almost all patients had positive thyrotropin receptor antibody (TRAb) or Thyroid stimulating immunoglobin (TSI) (96%, 55/57) except for two people who had imaging findings consistent with Graves’ disease (3%, 2/57). Thyroid stimulating hormone (TSH) levels were provided for 54 patients and they were decreased in all of them (100%, 54/54).

Thyroid ultrasound data was provided for 36 patients. Twenty-four of them had increased vascularity (67%, 24/36) (**Table 2**). Data regarding thyroid scintigraphy was provided for only 12 cases, with the majority having findings of increased diffuse uptake consistent with Graves’ disease (75%, 9/12). Data regarding treatment was available for 53 cases. Most of them received thionamides (47%, 25/53). The clinical status after treatment was provided for 37 patients and it was improved in the majority of them (84%, 31/37).

**Table 2:** Laboratory and imaging findings of the reported cases.

Author,Year,Country	Case number	Thyroid function tests	Normal references for thyroid function tests	Thyroid autoantibodies	Thyroidultrasound	Thyroid scintigraphy
Bostan, 2022, (38) Turkey	Case 1	TSH: < 0.01 mIU/L	0.27–4.2 mIU/L	•TRAb: 12.18 IU/L •TSI: NA •Anti-TPO:284 IU/ml •Anti-Tg:119 IU/ml	Hypoechoic areas, increased vascularity in a ‘Thyroid inferno’ pattern	NA
		FT3: 9.65 ng/L	2–4.4 ng/L		
		T3:NA	NA		
		FT4: 2.67 ng/dL	0.93–1.7 ng/dL		
	Case 2	TSH<0.01 mIU/L	0.27–4.2 mIU/L	•TRAb: 3.01 IU/L •TSI: NA •Anti-TPO:435 IU/ml •Anti-Tg:236 IU/ml	Increased vascularity	NA
		FT3: 13.50 ng/L	2–4.4 ng/L	
		T3: NA	NA	
		FT4:3.86 ng/dL	0.93–1.7 ng/dL	
	Case 3	TSH: <0.01 mIU/L	0.27–4.2 mIU/L	•TRAb: 19.30 IU/L •TSI: NA •Anti-TPO: 325 IU/ml •Anti-Tg:11 IU/ml	Increased vascularity	NA
		FT3: 21.70 ng/L	2–4.4 ng/L
		T3: NA	NA
		FT4: 7.77 ng/dL	0.93–1.7 ng/dL
	Case 4	TSH: <0.01 mIU/L	0.27–4.2 mIU/L	•TRAb: 17.84 IU/L •TSI: NA •Anti-TPO: 55 IU/ml •Anti-Tg: 1197 IU/ml	Normal thyroid gland size, highly heterogeneous parenchyma, increased vascularity	Increased diffuse activity uptake in both thyroid lobes
		FT3: 8.83 ng/L	2–4.4 ng/L
		T3: NA	NA
		FT4: 4.01 ng/dL	0.93–1.7 ng/dL
	Case 5	TSH: <0.01 mIU/L	0.27–4.2 mIU/L	•TRAb: 5.04 IU/L •TSI: NA •Anti-TPO: 12.4 IU/ml •Anti-Tg: 18.2 IU/ml	Enlarged thyroid with multinodular goiter	Hypoactive multinodular hyperplasic thyroid gland
		FT3: 12.6 ng/dl	2–4.4 ng/L	
		T3: NA	NA	
		FT4: 3.72 ng/dL	0.93–1.7 ng/dL	
	Case 6	TSH: <0.01 mIU/L	0.27-4.2mIU/L	•TRAb: 22.74 IU/L •TSI: NA •Anti-TPO:11.2 IU/ml •Anti-Tg: 320 IU/ml	Diffuse hypoechoic areas in the bilaterally enlarged thyroid gland and increased vascularity	NA
		FT3: 11.0 ng/dL	2-4.4 ng/dL	
		T3: NA	NA	
		FT4: 3.32 ng/dL	0.93-1.7 ng/dL	
	Case 7	TSH: <0.01 mIU/L	0.27-4mIU/L	•TRAb: 9.10 IU/L •TSI: NA •Anti-TPO: 146 IU/ml •Anti-Tg: 334 IU/ml	Diffuse hypoechoic areas in the bilaterally enlarged thyroid gland and increased vascularity in a ‘Thyroid inferno’ pattern	NA
		FT3: 25.3 ng/L	2-4.4 ng/L	
		T3: NA	NA	
		FT4: 7.7 ng/dL	0.93-1.7 ng/L	
Chees, 2022, (39) Singapore	Case 1	TSH: 0.01 mIU/L	0.45-4.5 mIU/L	•TRAb: 7.3IU/L* •TSI: NA •Anti-TPO: NA •Anti-TG: NA	NA	NA
		FT3: NA	NA
		T3: NA	NA
		FT4: 45 pmol/L	8-16 pmol/L
	Case 2	TSH: <0.01 mIU/L	0.45-4.5mIU/L	•TRAb: 3.8 IU/ml* •TSI: NA •Anti-TPO: NA •Anti-TG: NA	NA	NA
		FT3: NA	NA	
		T3: NA	NA	
		FT4: 60 pmol/L	8-16 pmol/L	
	Case 3	TSH: 0.01 mIU/L	0.45-4.5 mIU/L	•TRAb: 11.2 IU/ml* •TSI: NA •Anti TPO: NA •Anti-TG: NA	NA	NA
		FT3: 23.8 pmol/L	3.5-6 pmol/L	
		T3: NA	NA	
		FT4: 68 pmol/L	8-16 pmol/L	
	Case 4	TSH: <0.01 mIU/L	0.45-4.5 mIU/L	•TRAb: 32 IU/ml* •TSI: NA •Anti-TPO: NA •Anti-TG: NA	NA	NA
		FT3: NA	NA	
		T3: NA	NA	
		FT4: 29 pmol/L	8-16 pmol/L	
	Case 5	TSH: <0.01 mIU/L	0.45-4.5mIU/L	•TRAb: 4.6 IU/ml* •TSI: NA •Anti-TPO: NA •Anti-TG: NA	NA	NA
		FT3: NA	NA	
		T3: NA	NA	
		FT4: 29 pmol/L	8-16 pmol/L	
	Case 6	TSH: <0.01 mIU/L	0.45-4.5 mIU/L	•TRAb: 6.2 IU/ml* •TSI: NA •Anti-TPO: NA •Anti-TG: NA	NA	NA
		T3: NA	3.5-6 pmol/L	
		FT3: >40 pmol/L	NA	
		FT4: 70 pmol/L	8-16 pmol/L	
	Case 7	TSH: <0.01	0.45-4.5 mIU/L	•TRAb: 12.8 IU/ml* •TSI: NA •Anti-TPO: NA •Anti-TG: NA	NA	NA
		FT3: NA	3.5-6 pmol/L	
		T3: NA	NA	
		FT4: 49 pmol/L	8-16 pmol/L	
	Case 8	TSH: 0.02 mIU/L	0.45-4.5 mIU/L	•TRAb: 6.2 IU/ml* •TSI: NA •Anti-TPO: NA •Anti-TG: NA	NA	NA
		FT3: NA	3.5-6 pmol/L	
		T3: NA	NA	
		FT4: 14 pmol/L	8-16 pmol/L	
	Case 9	TSH: 0.02 mIU/L	0.45-4.5 mIU/L	•TRAb: 2.9 IU/ml* •TSI: NA •Anti-TPO: NA •Anti-TG: NA	NA	NA
		FT3: 6.3 pmol/L	3.5-6 pmol/L	
		T3: NA	NA	
		FT4: 15 pmol/L	8-16 pmol/L	
	Case 10	TSH: 0.01 mIU/ml	0.45-4.5 mIU/L	•TRAb: 3.9 IU/ml* •TSI: NA •Anti-TPO: NA •Anti-TG: NA	NA	NA
		FT3: NA	3.5-6 pmol/L	
		T3: NA	NA	
		FT4: 20 pmol/L	8-16 pmol/L	
	Case 11	TSH: 0.01 mIU/ml	0.45-4.5 mIU/L	•TRAb: 2.4 IU/ml* •TSI: NA •Anti-TPO: NA •Anti-TG: NA	NA	NA
		FT3: NA	3.5-6 pmol/L	
		T3: NA	NA	
		FT4: 20 pmol/L	8-16 pmol/L	
	Case 12	TSH: 0.01 mIU/L	0.45-4.5 mIU/L	•TRAb: 5.8 IU/ml* •TSI: NA •Anti-TPO: NA •Anti-TG: NA	NA	NA
		FT3: >40 pmol/L	3.5-6 pmol/L	
		T3: NA	NA	
		FT4: 70 pmol/L	8-16 pmol/L	
Chua, 2022, (37) Singapore	Case 1	TSH: <0.01 mIU/L	0.7-4.28 mIU/L	•TRAb: 3.85 IU/L^**^ •TSI: NA •Anti-TPO: NA •Anti-TG: NA	NA	NA
		FT3: NA	NA
		T3: NA	NA
		FT4: 48.2 pmol/L	12.7-20.3 pmol/L
	Case 2	TSH: <0.005 mIU/L	0.7-4.28 mIU/L	•TRAb: 5.75 IU/L^**^ •TSI: NA •Anti-TPO: 0.3 IU/ml^†^ •Anti-TG: NA	Heterogeneous thyroid gland with increased vascularity, a few sub-centimetre solid and cystic nodules	NA
		FT3: NA	NA	
		T3: NA	NA	
		FT4: 45.1 pmol/L	12.7-20.3 pmol/L	
Di Filippo, 2021, Italy	Case 1	TSH:0.005 uIU/mL	NA	•TRAb: 7.9 IU/L^***^ •TSI: NA •Anti TPO: NA •Anti Tg: NA	Gland enlargement with pseudonodules, increased vascularity	NA
		FT3: 7.9 pg/ml	2-4.4 pg/ml
		T3: NA	NA
		FT4: 2.96 ng/dL	0.6-1.12 ng/dL
	Case 2	TSH: <0.004 uIU/mL	NA	•TRAb:3.2 IU/L^***^ •TSI: NA •Anti TPO: NA •Anti Tg: NA	Gland enlargement, increased vascularity	NA
		FT3: NA	2-4.4 pg/ml
		T3: NA	NA
		FT4: 4.96 ng/dL	0.6-1.12 ng/dL
Goblirsch, 2021, (23) USA	Case 1	TSH: <0.02 IU/mL	0.35-2 IU/mL	•TRAb: NA •TSI: 347% •Anti TPO: 8.9 IU/mL^†^ •Anti Tg: NA	Multinodular disease	NA
		FT3: NA	FT3: NA	
		T3: 5.3 ng/mL	0.8-2.8 ng/mL	
		FT4: 7.2 ng/dL	0.9-1.7 ng/dL	
Hamouche, 2021, (25) USA	Case 1	TSH: <0.005 uIU/mL	0.282-4 uIU/mL	•TRAb: NA •TSI: 200%^‡^ •Anti TPO: 119 IU/mL •Anti Tg: 53^§^	Heterogeneous thyroid with underlying micronodules suggestive of thyroiditis.	72% homogeneous uptake
		FT3: NA	NA
		T3: 397 ng/dL	69-154 ng/dL
		FT4: 5.41 ng/d	0.84-1.62 ng/dL
Lee, 2021,(41) South Korea	Case 1	TSH: 0.010 IU/mL	0.55-4.78 IU/mL	•TRAb: 6.42 IU/L^**^ •TSI: NA •Anti TPO: 77.72 IU/ml •Anti Tg: 137.5 IU/ml	Increased vascularity	NA
		FT3: NA	NA
		T3: NA	NA
		FT4: 33.92 ng/dL	11.5-22.7 ng/dL
	Case 2	TSH: <0.008 IU/mL	0.55-4.78 IU/mL	•TRAb: 6.1 IU/L^**^ •TSI: NA •Anti TPO: 43.3 IU/ml •Anti Tg: NA	Increased vascularity	NA
		FT3: NA	NA
		T3: NA	NA
		FT4: 73.80 ng/dL	11.5-22.7 ng/dL
	Case 3	TSH: <0.012 IU/mL	0.55-4.78 IU/mL	•TRAb: 2.9 IU/L^**^ •TSI: NA •Anti TPO: <15 IU/ml •Anti Tg: 295.5 IU/ml	Diffuse goiter with ill-defined hypoechoic lesion	NA
		FT3: NA	NA
		T3: NA	NA
		FT4: 36.98 ng/dL	11.5-22.7 ng/dL
	Case 4	TSH: <0.008 IU/mL	0.55-4.78 IU/mL	•TRAb: 4.24 IU/L^**^ •TSI: NA •Anti TPO: NA •Anti Tg: NA	Increased vascularity	NA
		FT3: NA	NA
		T3: NA	NA
		T4: 26.61 ng/dL	11.5-22.7 ng/dL
Lui, 2021, (26) China	Case 1	TSH: <0.02 mIU/L	0.47-4.68 mIU/L	•TRAb: NA •TSI: 420% •Anti TPO: NA •Anti Tg: NA	Heterogeneous thyroid echogenicity with increased vascularity	Diffuse markedly increased uptake over both lobes, increased blood flow
		FT3: 30.5 pmol/L	4.26-8.1 pmol/L
		T3: NA	NA
		FT4: 66.6 pmol/L	10-28.2 pmol/L
Oguz, 2021, (36) Turkey	Case 1	TSH: <0.015 mIU/L	0.38-5.33 mIU/L	•TRAb: 10.3 IU/mL •TSI: NA •Anti TPO: 195.7 IU/mL^†^ •Anti Tg: 7.1 IU/mL^§§^	Diffuse hyperplasia, increased vascularity	Diffusely increased radiotracer uptake
		FT3: 8.79 pmol/L	3.8-6 pmol/L
		T3: NA	NA
		FT4: 27.92 pmol/L	7.86-14.41 pmol/L
	Case 2	TSH: <0.0015 mIU/L	0.38-5.33 mIU/L	•TRAb: 0.97 IU/mL •TSI: NA •Anti TPO: 0.7 IU/mL^†^ •Anti Tg<-0.9 IU/mL^§§^	Diffuse hyperplasia, increased vascularity	24-hour RAIU: 27%
		FT3: 7.19 pmol/L	3.8-6 pmol/L
		T3: NA	NA
		FT4: 12.15 pmol/L	7.86-14.41 pmol/L
	Case 3	TSH: 0.015 mIU/L	0.38-5.33 mIU/L	•TRAb: 0.25 IU/mL •TSI: NA •Anti TPO: 0.8IU/mL^†^ •Anti Tg: 1.8 IU/mL^§§^	Diffuse hyperplasia, increased vascularity	24-hour RAIU: 61%
		FT3: 11 pmol/L	3.8-6 pmol/L
		T3: NA	NA
		FT4: 33.1 pmol/L	7.86-14.41 pmol/L
	Case 4	TSH: 0.01 mIU/L	0.38-5.33 mIU/L	•TRAb: 1.9 IU/mL •TSI: NA •Anti TPO: 196 IU/mL^†^ •Anti Tg: 167 IU/mL^§§^	Diffuse hyperplasia, increased vascularity	24-hour RAIU: 23%
		FT3: 7.8 pmol/L	3.8-6 pmol/L
		T3: NA	NA
		FT4: 25.5 pmol/L	7.86-14.41 pmol/L
	Case 5	TSH: 0.0 mIU/L	0.38-5.33 mIU/L	•TRAb: 3 IU/mL •TSI: NA •Anti TPO: 1.2 IU/mL^†^ •Anti Tg<0/9 IU/mL^§§^	NA	24-hour RAIU 39%
		FT3: 10.54 mIU/L	3.8-6 pmol/L
		T3: NA	NA
		FT4: 31.65 pmol/L	7.86-14.41 pmol/L
Patrizio, 2021, (30) Italy	Case 1	TSH: <0.004 mIU/L	0.4–4.00 mIU/L	•TRAb: 6.48 IU/L •TSI: NA •Anti TPO: 21 IU/mL^†^ •Anti Tg: 30 IU/mL^§§§^	Enlarged thyroid gland with heterogeneous echotexture, increased vascularity	NA
		FT3: 15 ng/dL	2.7–5.7 ng/L	
		T3: NA	NA	
		FT4: 5.56 ng/dL	0.7–1.7 ng/dL	
Pierman, 2021, (29) Belgium	Case 1	TSH: 0.01 mIU/L	0.4-2.75 mIU/L	•TRAb: >40 IU/L^****^ •TSI: NA •Anti TPO: NA •Anti Tg: NA	NA	NA
		FT3: 22.09 pmol/L	3-6.5 pmol/L
		T3: NA	NA
		FT4: 2.54 ng/dL	0.75-1.6 ng/dL
Pla Peris, 2022, (22) Spain	Case 1	TSH: <0.005 mUI/L	0.38-5.33 mUI/L	•TRAb: 3.6 U/L •TSI: NA •Anti TPO: 30 U/ml^†^ •Anti Tg: <0.9U/ml^§§^	Enlarged thyroid, increased vascularity	Diffuse markedly increased uptake over both lobes
		FT3: NA	NA
		T3: NA	NA
		FT4: 2.3 ng/dL	0.54-1.24 ng/dL
	Case 2	TSH: <0.005 mUI/L	0.38-5.33 mUI/L	•TRAb: 4.39 U/L •TSI: NA •Anti TPO: NA •Anti Tg: 2.5 U/ml^§§^	Enlarged thyroid, increased vascularity	Diffuse markedly increased uptake over both lobes
		FT3: NA	NA
		T3: NA	NA
		FT4: 2.9 ng/dL	0.54-1.24 ng/dL
	Case 3	TSH: <0.005 mUI/L	0.38-5.33 mUI/L	•TRAb: 5.1 U/L •TSI: NA •Anti TPO: 30 U/ml^†^ •Anti Tg: 55 U/ml^§§^	Enlarged thyroid, increased vascularity	NA
		FT3: NA	NA
		T3: NA	NA
		FT4: 4.7 ng/dL	0.54-1.24 ng/dL
	Case 4	TSH: <0.005 mUI/L	0.38-5.33 mUI/L	•TRAb: 3.2 U/L •TSI: NA •Anti TPO: 60 U/ml^†^ •Anti Tg: 90 U/ml^§§^	Enlarged thyroid, increased vascularity	NA
		FT3: NA	NA
		T3: NA	NA
		FT4: 4.2 ng/dL	0.54-1.24 ng/dL
	Case 5	TSH: <0.005 mUI/L	0.38-5.33 mUI/L	•TRAb: 3.8 U/L •TSI: NA •Anti TPO: <0.5 U/ml^†^ •Anti Tg: 0.9 U/ml^§§^	NA	NA
		FT3: NA	NA
		T3: NA	NA
		FT4: 1.8 ng/dL	0.54-1.24 ng/dL
Pujol, 2021, (27) Spain	Case 1	TSH: <0.001 μIU/mL	0.35-4.95 μIU/mL	•TRAb: 12.54 IU/ml •TSI: 12.54 IU/ml^‡‡^ •Anti TPO: 3303.7 IU/ml^††^ •Anti Tg: 36.57^§§^	Diffuse decrease in echogenicity with some echogenic septum, increased vascularity	NA
		FT3: 7.46 pg/mL	1.58-3.91 pg/mL	
		T3: NA	NA	
		FT4: 2.01 ng/dL	0.7-1.48 ng/dL	
Raven, 2021, (40) Australia	Case 1	TSH: < 0.02 mIU/L	0.5-4.0 mIU/L	•TRAb: NA •TSI: 24 IU/ml •Anti TPO: > 1300 IU/ml •Anti Tg: 33 IU/ml	Diffusely heterogeneous thyroid, increased vascularity	NA
		FT3: > 30 pmol/L	3.5-6 pmol/L
		T3: NA	NA
		FT4: 64 pmol/L	10-20 pmol/L
Shih, 2022, (42) Taiwan	Case 1	TSH: <0.0038 mIU/L	0.35-4.94 mIU/L	•TRAb: 42.4%^*****^ •TSI: NA •Anti TPO: 64.58 IU/ml ^††^ •Anti-Tg: <3 IU/ml^§§§§^	NA	NA
		FT3: NA	NA
		T3: NA	NA
		FT4: 1.29 ng/dL	0.7-1.48 ng/dL
	Case 2	TSH: 0.0091 mIU/L	0.35-4.94 mIU/L	•TRAb: 68.7%^*****^ •TSI: NA •Anti TPO<3 IU/mL^††^ •Anti-Tg: 1494.78IU/mL^§§§§^	NA	NA
		FT3: NA	NA
		T3: NA	NA
		FT4: 1.06 ng/dL	0.7-1.48 ng/dL
	Case 3	TSH<0.0038 mIU/L	0.35-4.94 mIU/L	•TRAb: 80.9%^*****^ •TSI: NA •Anti TPO: 206.64<3 IU/mL^††^ •Anti-Tg: 2904.39 IU/mL^§§§§^	NA	NA
		FT3: NA	NA	
		T3: NA	NA	
		FT4: 0.83 ng/dL	0.7-1.48 ng/dL	
Sriphrapradang, 2021 (I), (31) Thailand	Case 1	TSH: <0.0036 mIU/L	0.35-4.94 mIU/L	•TRAb: 3.23 IU/ml •TSI: NA •Anti TPO: NA •Anti Tg: NA	NA	NA
		FT3: >20 pg/mL	1.88–3.18 pg/mL	
		T3: NA	NA	
		FT4: 3.19 ng/dL	0.7–1.48 ng/dL	
Sriphrapradang, 2021 (II), (32) Thailand	Case 1	TSH: 0.006 mIU/L	0.35-4.94 mIU/L	•TRAb: 13.4 IU/ml •TSI: NA •Anti TPO: NA •Anti Tg: NA	NA	NA
		FT3: 3.21 pg/mL	1.88–3.18 pg/mL	
		T3: NA	NA	
		FT4: 1.29 ng/dL	0.7–1.48 ng/dL	
Vera- Lastra, 2021, (34) Mexico	Case 1	TSH: <0.001 μgUi/mL	0.27-4.4 μgUi/mL	•TRAb: 16.56 IU/ml •TSI: 380% •Anti TPO: 3405 IU/ml^††^ •Anti Tg: 210 IU/ml^§^	NA	NA
		FT3: 10.5 pg/mL	2.04-4.4 pg/mL	
		T3: 251 ng/dL	64-181 ng/dL	
		FT4: 3.57 ng/d	0.93-1.71 ng/dL	
	Case 2	TSH: <0.001 μgUi/mL	0.27-4.4 μgUi/mL	•TRAb: 5.85 IU/ml •TSI:NA •Anti TPO: 833 IU/ml^††^ •Anti Tg: 33 IU/ml^§^	NA	NA
		FT3: 9.2 pg/mL	2.04-4.4 pg/mL	
		T3: 216 ng/dL	64-181 ng/dL	
		FT4: 1.84 ng/d	0.93-1.71 ng/dL	
Weintraub, 2021, (24) USA	Case 1	TSH: <0.008	0.45-4.5 μIU/ml	•TRAb: 32 IU/L •TSI: >40 •Anti TPO: 1730 IU/ml^†^ •Anti Tg: NA	Heterogeneous, hypervascular, enlarged gland	NA
		FT3: NA	NA	
		T3: 10.3 nmol/L	0.9-2.8 nmol/L	
		FT4: 108 pmol/L	10.6-22.8 pmol/L	
	Case 2	TSH: 0.011 μIU/ml	0.45-4.5 μIU/ml	TRAb: 22 IU/L •TSI: NA •Anti TPO: 1149 IU/ml^†^ •Anti Tg: NA	Heterogeneous, hypervascular gland	Diffuse increased activity
		FT3: NA	NA
		T3: 4.6 nmol/L	0.9-2.8 nmol/L
		FT4: 30.9 pmol/L	10.6-22.8 pmol/L
	Case 3	TSH: 0.005 μIU/ml	0.45-4.5 μIU/ml	•TRAb: NA •TSI: NA •Anti TPO: 15 IU/ml† •Anti Tg: NA	NA	NA
		FT3: NA	NA
		T3: 2.5 nmol/L	0.9-2.8 nmol/L
		FT4: 22.9	10.6-22.8 pmol/L
Yamamoto, 2021, (28) Japan	Case 1	TSH: <0.008 mIU/mL	NA	•TRAb: 33.8 IU/L •TSI: NA •Anti TPO: NA •Anti Tg: NA	Goitre lesions	NA
		FT3: 23.2 ng/dL	NA	
		T3: NA	NA	
		FT4: 3.3 ng/dL	NA	
Zettinig, 2021, (33) Austria	Case 1	TSH: NA	NA	•TRAb: 4.2 •TSI: NA •Anti TPO: NA •Anti Tg: NA	NA	NA
		FT3: 11.10 pg/mL	2.15–4.12 pg/mL
		T3: NA	NA
		FT4: 3.56 ng/dL	0.70–1.70 ng/dL
	Case 2	TSH: NA	NA	•TRAb: 2.9 •TSI: NA •Anti TPO: NA •Anti Tg: NA	NA	NA
		FT3: 5.18 pg/mL	2.15–4.12 pg/mL
		T3: NA	NA
		FT4: 1.63 ng/dL	0.70–1.70 ng/dL

Ab, Antibodies; Anti Tg, Antithyroglobulin; RAIU, radioactive iodine uptake test; TRAb, thyroid receptor antibody; TSI, thyroid stimulating immunoglobulin; TSH, thyroid stimulating hormone; TPO, Thyroid peroxidase; NA, not available.

Normal range: TRAb <1.5 IU/L, *<1 IU/L, ** <1.75 IU/L, ***<2.9IU/L, ****<0.55 IU/L, *****<10%.

Anti TPO: 0–34 IU/ml, † <9 IU/ml, †† 0-5.6 IU/ml.

Anti-TG: 0–115 IU/ml, § <40 IU/mL, §§ <4 IU/mL, §§§ 0-30 IU/ml, § § § § <14.4 IU/ml.

TSI<140%, ‡<125%, ‡‡<0.7 IU/ml, ‡‡‡ <0.55 IU/ml.

### Quality of the studies

The mean quality score indicated that the studies reported on average 10 of the recommended 12 elements, defined by the guidelines. Only 3 studies had a perfect score of 12 while the second most common score was 11. The most frequently missing information was the following: adverse events after vaccine administration (76%, 16/21) (**Table 3**).

**Table 3:** Quality assessment of the included studies.

**Author, year**	**Q1**	**Q2**	**Q3**	**Q4**	**Q5**	**Q6**	**Q7**	**Q8**	**Q9**	**Q10**	**Q11**	**Q12**	**Overall**
Bostan, 2022 (38)	●	●	●	●	●	●	●	●	●	●	○	●	11
Chee, 2022 (39)	●	●	●	●	●	○	●	●	●	●	○	●	10
Chua, 2022 (37)	●	●	●	●	●	○	●	●	●	●	●	●	11
Di Fillipo, 2021 (35)	●	●	●	●	●	●	●	●	●	●	●	●	12
Goblirsch, 2021 (23)	●	●	●	●	●	●	●	●	●	●	○	●	11
Hamouche, 2021 (25)	●	●	●	●	●	●	●	●	●	●	●	●	12
Lee, 2021 (41)	●	●	●	○	●	○	●	●	●	●	○	●	9
Lui, 2021 (20)	●	●	●	●	●	●	●	●	●	●	●	●	12
Oguz, 2022 (36)	●	●	●	●	●	○	●	●	●	●	○	●	10
Patrizio, 2021 (30)	●	●	●	●	●	●	●	●	●	●	○	●	11
Pierman, 2021 (29)	●	●	●	●	●	○	●	●	●	●	○	●	10
Pla Pleris, 2022 (22)	●	●	●	○	●	○	●	○	●	●	○	●	8
Pujol, 2021 (27)	●	●	●	●	●	○	●	●	●	●	○	●	10
Raven, 2021(40)	●	●	●	●	●	○	●	●	●	●	○	●	10
Shih, 2022 (42)	●	●	●	○	●	○	●	○	●	○	○	●	7
Sriphrapradang, 2021 (31 (I)	●	●	●	○	●	●	●	●	●	●	○	●	10
Sriphrapradang, 2021 (32 (II)	●	●	●	●	●	●	●	●	●	●	○	●	11
Vera- Lastra, 2021(34)	●	●	●	●	●	○	●	○	●	●	○	●	9
Weintraub, 2021(24)	●	●	●	○	●	●	●	●	●	●	○	●	10
Yamamoto, 2021(28)	●	●	●	●	●	●	●	●	●	●	●	●	12
Zettining, 2021(33)	●	●	●	●	●	○	●	●	●	○	○	●	9

Q1 ,Appropriate title; Q2, Patient demographics; Q3, Current health status; Q4, Medical History; Q5, Physical examination; Q6, Patient disposition; Q7, Drug Identification; Q8, Dosage; Q9, Administration; Q10, Drug-reaction interface; Q11, Adverse events; Q12, Discussion ● = 1; ○ = No.

## Discussion

COVID-19 vaccine administration has not been considered a triggering factor for thyroid autoimmune disorders. However, emerging evidence, mainly from case reports and case series, suggests a potential association between COVID-19 vaccination and the development or recurrence of thyroid dysfunction including Graves’ disease. In our systematic review, we comprehensively examined the currently available literature to provide an overview of the reported cases of Graves’ disease following vaccination against SARS-CoV-2. Our study included 21 reports, which comprised 57 patients, in which Graves’ disease was reported after the administration of different COVID-19 vaccines. The onset of the symptoms started after administration of the first dose in most cases and clinical improvement was reported for the majority of patients.

### Results in the context of the literature

Graves’ disease is an autoimmune disorder most commonly presenting with hyperthyroidism and seropositivity for autoantibodies against the thyrotropin receptor (43–45). TRAb production is secondary to a Th1 immune response in which T cells react with peptides derived from thyroid autoantigens leading to increased secretion of autoantibodies from B cells. TRAb stimulates thyroid hormone synthesis, which leads to thyroid growth and diffuse goiter. Multiple precipitating factors have been proposed including female gender, genetic predisposition, stress, smoking, medication, iodine, pregnancy and infection. Several cases of Graves’ disease have been reported following COVID-19 infection with the T cell sensitization to the TSH receptor antigen being proposed as the driving mechanism in people with genetic predisposition (45). Specifically, in a systematic review, Tutal et al. reported 14 cases of Graves’ disease post COVID-19 infection (45).

Apart from COVID-19 infection, our study showed that COVID-19 vaccination may potentially be associated with Graves’ disease although evidence is still inconclusive. Following the sex distribution reported in the literature (46), Graves’ disease post vaccination presented most commonly in females (75%) with palpitations and weight loss. Overall, 19 people had a pre-existing thyroid disorder such as multinodular goiter, Graves’ disease, autoimmune thyroiditis or subclinical hypothyroidism. Interestingly, most patients with background thyroid dysfunction had received an mRNA vaccine. Regrettably, the impact of previous COVID-19 infection could not be assessed considering the lack of data in the majority of cases but remains a possibility. Based on the short interval between vaccination and initiation of symptoms, Graves’ disease might have preceded vaccination on certain occasions. As expected, most cases were treated with thionamides and beta blockers. Steroids were used only in three cases for the amelioration of symptoms by reducing the conversion of T4 to T3. Although steroids consist one of the main therapeutic approaches in people with subacute thyroiditis, more concrete instructions on their use in Graves’ disease are needed considering their potential impact on the immune response triggered by vaccination.

Two reviews have attempted to present the evidence on thyroid dysfunction and COVID-19 vaccination so far. Caironi et al. and Jafarzadeh et al. included 29 and 21 number of patients with Graves’ disease respectively (47, 48). Our study focused solely on Graves’ disease including 57 patients. Overall our findings were in agreement regarding presenting symptoms, onset of symptoms post-vaccination and management. Distribution on different vaccine types was also similar.

Although the exact mechanism behind the potential association between COVID-19 vaccination and Graves’ disease remains to be elucidated, several theories have been suggested. Autoimmune/inflammatory syndrome induced by adjuvants (ASIA) is the most frequently cited theory (49). Adjuvants are used to increase immune response to the active substance and although essential for adequate immune system stimulation, they have been considered the etiological factor of ASIA following Hepatitis B and HPV immunization in the past most likely due to an intense immune response or genetic predisposition (50). This results from the formation of autoantibodies or systemic/localised inflammation, it rarely involves autoimmune thyroid disease and it’s most commonly reported within the first 3 weeks post vaccination (51). Although, mRNA vaccines do not use of adjuvants, they contain lipid nanoparticles which facilitate mRNA transport into cells and could potentially induce immune response in predisposed people (52). Additionally, the presence of the ACE-2 receptor in the thyroid gland could offer another explanation for the endocrine effects reported in individuals following the SARS-CoV-2 infection or vaccination since it constitutes the entry point of the virus into host cells (53). Cellular entry could lead to a direct inflammatory or immune mediated injury on thyroid cells with subsequent clinical manifestations (54). It is worth noting that the mRNA of ACE-2 receptor is also expressed in thyroid cells as confirmed by studies in thyroid tissue specimens and cultures, making them a potential target for viral entry (55, 56).

Another theory includes the possible effect of molecular mimicry in the development of autoimmune thyroid disorders (29). Thyroid peroxidase peptide sequences in thyroid tissue share similarities with the SARS-CoV-2 proteins, such as the spike protein that comprise a major target of the mRNA vaccines (57). It has been speculated that this could lead to cross-recognition between the modified SARS-CoV-2 spike protein encoded in the mRNA vaccine and the thyroid target proteins resulting in autoimmunity and it has been demonstrated that spike protein, nucleoprotein and membrane protein all cross-react with thyroid peroxidase (57). Additionally, cytokines such as Interferon gamma have been identified in both Graves’ disease and the SARS-CoV-2 infection (58). Results from a phase I/II vaccine candidate mRNA BNT162b1 suggest a Th1 type immune response involving interferon gamma, which could imply a modification of the cytokine environment that could favor the Th1 population and subsequently the production of autoantibodies (59).

### Strengths and limitations

Our study is the first to systematically review the association between COVID-19 vaccination and onset or exacerbation of Graves’ disease. Our findings present a comprehensive review of the currently available literature and highlight published data with rigorous quality assessment of included studies.

However, some limitations still persist. A broader drawback underlies the low-quality nature of case reports and case series included in our review, which affects the validity and scope of conclusions that can be reached. Specifically, the potential risk of bias of these studies is inevitable, as these are exposed to the risk of overinterpretation and selection bias. In this way, their reported data although interesting may be far from the truth without reflecting a valid description. Thus, causality cannot be inferred and requires insight from mechanistic studies.

## Conclusion

Although the currently available COVID-19 vaccines have established a safe profile and the benefits of vaccination outweigh the possible adverse events, patients can potentially experience mild to moderate side effects including thyroid related complications. Graves’ disease is possibly a condition physicians and other healthcare professionals may expect to see in patients receiving COVID-19 vaccines. While the above adverse event is rare, considering the scarcity of available data in scientific literature, and causality is not yet confirmed, the increased awareness of clinicians and the early recognition of the disorder is important for the optimal management of these patients.

## Data availability statement

The original contributions presented in the study are included in the article/Supplementary Material. Further inquiries can be directed to the corresponding author.

## Author contributions

Conceptualization, KKT, KSK; Methodology, KKT, KSK; Validation, KKT, DS, KSK; Investigation, KKT, KSK; Resources, KKT, DS, KSK; Writing—Original Draft Preparation, KKT, PG, DS, KSK; Writing—Review & Editing, KKT, DS, KSK; Visualization, KKT, DS, KSK; Supervision, DS, KSK; Project Administration, PG, DS, KSK. All authors have read and agreed to the published version of the manuscript.

## Conflict of interest

The authors declare that the research was conducted in the absence of any commercial or financial relationships that could be construed as a potential conflict of interest.

## Publisher’s note

All claims expressed in this article are solely those of the authors and do not necessarily represent those of their affiliated organizations, or those of the publisher, the editors and the reviewers. Any product that may be evaluated in this article, or claim that may be made by its manufacturer, is not guaranteed or endorsed by the publisher.
